# Recent advances in cytomegalovirus infection management in solid organ transplant recipients

**DOI:** 10.1097/MOT.0000000000001139

**Published:** 2024-01-30

**Authors:** Paolo Antonio Grossi, Maddalena Peghin

**Affiliations:** Infectious and Tropical Diseases Unit, Department of Medicine and Surgery, University of Insubria-ASST-Sette Laghi, Varese, Italy

**Keywords:** cell mediated immunity, cytomegalovirus, letermovir, maribavir, solid organ transplant

## Abstract

**Purpose of review:**

Human cytomegalovirus (CMV) continues to be the most important infectious complication following solid organ transplantation (SOT).

**Recent findings:**

Universal prophylaxis and preemptive therapy are the most adopted strategies for prevention of CMV disease globally. Prophylaxis with valganciclovir is the most widely used approach to CMV prevention, however leukopenia and late onset CMV disease after discontinuation of prophylaxis requires new strategies to prevent this complication. The use of assays detecting CMV-specific T cell-mediated immunity may individualize the duration of antiviral prophylaxis after transplantation. Letermovir has been recently approved for prophylaxis in kidney transplant recipients. CMV-RNAemia used together with CMV-DNAemia in the viral surveillance of CMV infection provides accurate information on viral load kinetics, mostly in patients receiving letermovir prophylaxis/therapy. The development of refractory and resistant CMV infection remains a major challenge and a new treatment with maribavir is currently available. In the present paper we will review the most recent advances in prevention and treatment of CMV diseases in SOT recipients.

**Summary:**

Recent findings, summarized in the present paper, may be useful to optimize prevention and treatment of CMV infection in SOT.

## INTRODUCTION

Human cytomegalovirus (CMV) continues to be the most important infectious complication following solid organ transplantation (SOT), where it may cause adverse outcomes for allograft and recipient survival due to significant number of direct and indirect effects, including CMV disease, drug-related toxicities, bacterial and opportunistic superinfections and graft rejection. Moreover, it may increase the cost of transplantation, and negatively impact SOT quality of life [1,2^▪▪^]. However, there are still several unmet needs in CMV management in posttransplant settings [3]. In the present paper we will review the most recent advances in prevention and treatment of CMV diseases in SOT recipients. 

**Box 1:**
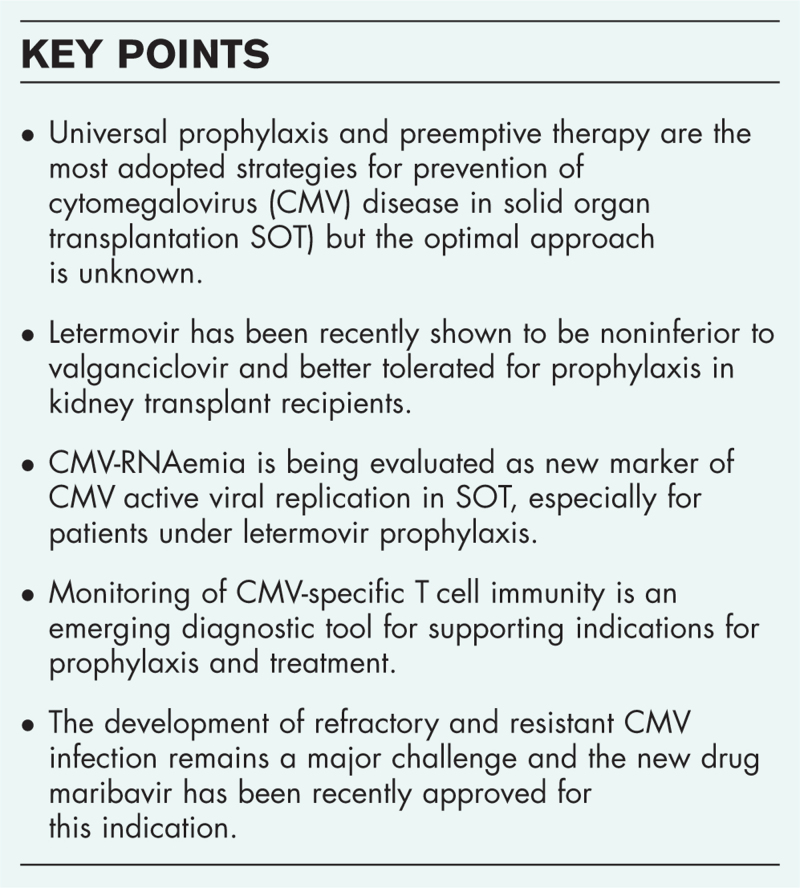
no caption available

## ADVANCES IN CYTOMEGALOVIRUS DIAGNOSIS

### Cytomegalovirus detection

CMV-DNAemia with quantitative real-time polymerase chain reaction test is the major tool for posttransplant monitoring of viral replication, diagnosis of active disease, check for response to antiviral therapy, and risk of relapse detection or antiviral resistance. However, variability in the results persists due to differences in sample types (plasma or whole blood), PCR assay platforms, and different quantification standards used in laboratories worldwide, despite the introduction of international standards by the World Health Organization [4^▪▪^]. Of interest that CMV DNA cut-off values for preemptive therapy are still a matter of debate and may vary according to guidelines, monitoring techniques, and transplant centers (range 10–10 000 copies/ml in plasma and 5–100 000 copies/ml in whole blood) [4^▪▪^].

However, conventional CMV-DNAemia might be an inappropriate method of CMV monitorization during letermovir prophylaxis because of overestimation of the viral load and underestimation of treatment success. Indeed, letermovir inhibits CMV replication at a later stage compared to conventional DNA polymerase inhibitors, leading to the production of nonviable virions and CMV-DNAemia by Real-time quantitative PCR (RT-qPCR) is not able to distinguish between viable and nonviable virus. CMV-RNAemia is a new diagnostic tool that is being evaluated as new marker of CMV active viral replication in SOT [5]. Of interest, that CMV-RNAemia, combined with CMV-DNAemia has been studied to detect active infection and to guide therapy during posttransplant period. It has been observed that CMV-RNAemia may provide accurate information on viral load kinetics, mostly in patients receiving letermovir prophylaxis or therapy [6].

### Cytomegalovirus resistance testing

It is recommended to perform CMV drug resistance testing by automated genotypic resistance investigation directly from whole-blood or plasma specimens, which are more reliable with positive viral-load values of at least 1000 copies/ml [7].

Newly mapped mutations and their phenotypes provide more detail on CMV cross-resistance properties. Mutations on the UL97 phosphotransferase gene give resistance to ganciclovir, whereas mutation on UL54 polymerase gene confers resistance to one or all of the CMV DNA polymerase inhibitors (ganciclovir, foscarnet and cidofovir). As regards advances in CMV drug resistance, mutation maps have been recently updated with current information for the terminase inhibitor letermovir (UL56 mutations) and the UL97 kinase inhibitor maribavir (pUL97 mutations) [7]. Therefore it is recommended to test patients with refractory CMV for basic CMV resistance-gene studies, including UL54, UL97, and UL56 mutations on the basis of drug exposure [2^▪▪^].

Performance of rapid next-generation whole genome sequencing on peripheral blood is another emerging technology allowing detection of virulence and new emerging antiviral resistance genes, as well as pathogen identification [8].

## CYTOMEGALOVIRUS SPECIFIC IMMUNITY

Donor and recipient CMV IgG is the only risk stratification test routinely performed. It is based on the principle that seronegative recipients receiving a seropositive graft (D+/R−) are at the highest risk of developing primary CMV infection, whereas seropositive patients (R+) are at an intermediate risk [1].

However, risk of posttransplantation CMV is more complex than just a CMV serostatus [9]. Cell-mediated immunity (CMI) against CMV (specific CD4^+^ and CD8^+^ T lymphocytes) is predominant in conferring protection against CMV-related disease [10^▪▪^]. Several new assays that assess measurement of CMV-CMI by using a stimulant to trigger immune cells, primarily T cells, and quantify the cytokine response to stimulation, have been developed with the aim to improve tailored strategies for treatment and prevention of CMV in SOT [11].

CMV-CMI monitoring is an emerging tool that has been evaluated with variable results (a) prior to transplantation to predict risk of CMV infection after transplantation; (b) to determine the optimal duration of antiviral prophylaxis and monitoring for viremia; (c) to decide on antiviral therapy when asymptomatic replication occurs; (d) to determine the need for secondary prophylaxis or predict risk of CMV recurrence, after completing treatment of CMV infection [10^▪▪^]. In general, high CMV-CMI predicts protection against CMV evolution whereas low CMV-CMI increases the likelihood of CMV replication or infection occurrence. Interestingly, patients with indeterminate CMV-CMI results suggest deeply annulled immunity or absence of CMV recognition [12]. However, most available literature is based on observational studies with limited interventional randomized trials and is mainly focused on kidney transplant recipients. Moreover assay-related differences in prediction of CMV infection or disease have been observed [10^▪▪^,^13^].

As regards pretransplant risk stratification, individuals with pretransplant CMV-CMI are at lower risk of developing CMV infection and of having severe CMV infection after SOT, manifesting with lower viral load and less invasive disease [14]. However, induction immunosuppression (especially of T-cell depleting therapies, such as Anti Thymocyte Globulin, ATG) and type of transplant could impact the predictive value of pretransplantation CMV-CMI [14].

Monitoring CMV-specific CMI soon after transplantation further defines the CMV infection prediction risk and might allow CMV-CMI guided versus fixed duration of antiviral prophylaxis against CMV in SOT [15–18]. In a recent multicenter randomized trial on kidney and liver SOT (D+/R− and R+ receiving ATG) in Switzerland CMV-CMI significantly reduced the use of antiviral prophylaxis, but the authors were unable to establish noninferiority of this approach on the co-primary outcome of clinically significant CMV infection [17].

It has been demonstrated across different SOT groups that patients without CMV-CMI at the end of prophylaxis have a consistent higher risk of late-onset CMV events and posttreatment relapse [19]. Moreover, CMV-CMI may predict evolution of asymptomatic viremia following prophylaxis discontinuation to spontaneous viral clearance (when positive) or the development of a CMV disease (when negative) [20].

The risk of recurrent CMV infection is estimated between 20% and 30% and has been observed to be higher in CMV seronegative patients, lung transplant recipients, patients with recent acute rejection and with prolonged CMV DNAemia despite therapy. Despite limited evidence to date, data support the clinical assessment of CMV-CMI testing at the end of treatment to guide decisions regarding the need for and duration of secondary prophylaxis in SOT that have recovered from posttransplantation CMV infection [12,21,22].

Lastly, there has been some interest in using CMV-specific immune monitoring assays to help decision on treatment duration and management of prophylaxis following antirejection therapy but, as things stand now, there are no data to sustain this use routinely.

New immunological markers such as Torque teno virus (TTV) viral load and immune-monitoring are being associated with CMV-CMI testing to improve risk stratification [23].

## NEW ANTIVIRALS

After a long period without licensing of new anti-CMV drugs, recent years have seen the approval of two novel antivirals for CMV prevention or treatment: letermovir and maribavir. Characteristics of both drugs are summarized in Table 1.

**Table 1:** New anti CMV drugs

Drug	Target dosage and formulation	Renal and hepatic impairment	Drug interactions adverse effects cons	Other herpesviruses	Labeled indications and off label use	Resistance, virological failure and relapse
Letermovir	UL56, UL51, UL89 terminase 480 mg IV/PO every 24 h 240 mg IV/PO every 24 h If cyclosporine is used at the same time.	• Oral: No dosage adjustment necessary • CrCl <50 mL/minute: use with caution IV formulation and closely monitor serum Creatinine due to potential accumulation of IV vehicle (hydroxypropyl betadex). • CrCl ≤10 ml/min or for dialysis: insufficient data for dosage recommendations • Mild or moderate hepatic impairment (Child-Pugh class A or B): No dosage adjustment necessary. • Severe hepatic impairment (Child-Pugh class C): use not recommended	-Relevant drug-drug interaction: may increase levels of immunosuppressants • Gastrointestinal • Cough • Headache • Peripheral edema	• Not active against other herpes viruses • Additional prophylaxis targeting HSV/VZV in D+/R – or R+ SOT is recommended	• CMV prophylaxis in HSCT recipients (R+) • CMV prophylaxis in high risk kidney SOT (D+/R-) • Not labeled for pediatric patients • Off-label use as salvage therapy and secondary prophylaxis.	• Low barrier of resistance • Resistance to letermovir due to mutations in UL56 • No cross resistance with CMV DNA polymerase inhibitors (ganciclovir, cidofovir, and foscarnet)
Maribavir	pUL97 kinase 400 mg PO every 12 h	• End-stage renal disease or dialysis: no dosage adjustments • Severe hepatic impairment (Child-Pugh class C): no dosage adjustments (not studied)	-Relevant drug-drug interaction: may increase levels of immunosuppressants • Dysegusia • Gastrointestinal • Fatigue • Neutropenia • Acute Kidney injury -Poor CNS penetration	• Not active against other herpes viruses (only in vitro for EBV) • Additional prophylaxis targeting HSV/VZV in D+/R – or R+ SOT is recommended	• Treatment of CMV infection/disease refractory (with or without genotypic resistance) to treatment with ganciclovir, valganciclovir, cidofovir, or foscarnet in adults and pediatric patients ≥12 years of age and weighing ≥35 • Off-label use for uncomplicated CMV DNAemia	• Risk of virologic failure due to resistance during and after treatment • Risk of virologic relapse 4–8 weeks after discontinuation of treatment; longer courses may be needed for maintaining CMV suppression • UL97 mutation that predicts resistance to ganciclovir has not been reported to confer cross-resistance to maribavir • Some maribavir pUL97 resistance-mutations confer cross-resistance to ganciclovir and valganciclovir

CNS, central nervous system; CrCl, creatinine clearance; D, donor; EBV, Epstein−Barr; HSV, herpes simplex virus; IV, intravenous; PO, per os; R, recipient; VZV, varicella zoster virus. ^∗^ Accumulation of IV vehicle, hydroxypropyl betadex.

Letermovir has a mechanism of action distinct from valganciclovir/ganciclovir and other CMV antiviral agents. It inhibits CMV replication by targeting the CMV DNA terminase complex, which is required for viral DNA processing and packaging, affecting production of genome unit lengths, and altering virion maturation. This viral terminase complex appears to be very CMV-specific and has a high activity against DNA polymerase inhibitors resistant strains [24].

In a recently published randomized controlled double blind double dummy phase 3 trial, letermovir was noninferior to valganciclovir for prophylaxis of CMV disease over 52 weeks and better tolerated for prophylaxis in kidney SOT with lower rates of leukopenia or neutropenia. Letermovir has been recently approved for prophylaxis thereof [25^▪▪^].

Letermovir is not recommended for treatment of CMV disease, due to low barrier for genotypic resistance [24]. Letermovir has been sporadically used as salvage therapy (before maribavir approval) for the treatment of refractory and resistant CMV with report of many clinical failures and on-treatment emergence of resistance, especially in the setting of CMV diseases with high viral loads [26–28]. Of note that use of letermovir as secondary prophylaxis has also been associated with high rates of failure [29].

Maribavir is an oral bioavailable benzimidazole riboside. Unlike other anti-CMV drugs, Maribavir has a unique mechanism of action targeting the viral kinase pUL97 and its natural substrates, which are involved in the DNA replication, encapsidation and viral capsid nuclear egress. Maribavir proved to be superior to investigator assigned treatment (IAT: valganciclovir/ganciclovir, foscarnet, or cidofovir) in a phase 3 open label randomized study (SOLSTICE trial) involving 211 SOT recipients with refractory CMV infections (with or without resistance) [30^▪▪^]. Of note that the effect of maribavir was higher both in patients with genotypic resistance to IAT and in patients without resistance mutations. However, significant attention should be paid to high rates of CMV recurrence and to the risk of resistance development during treatment and after discontinuation of treatment [30^▪▪^,^31^].

Interestingly, a recent retrospective chart review of a sub-cohort of patients from the SOLSTICE trial, overall mortality at 52 weeks postmaribavir treatment initiation was lower than that previously reported for similar populations treated with conventional therapies for CMV infection with or without resistance [32].

## PROPHYLAXIS VERSUS PRE-EMPTIVE TREATMENT

Universal prophylaxis and preemptive therapy are the most adopted strategies for prevention of CMV disease globally. CMV prophylaxis refers to the use of antivirals in all patients at increased risk of CMV reactivation, whereas preemptive therapy refers to the administration of antivirals only with evidence of CMV replication. The optimal approach to the prevention and treatment of infection due to CMV remains uncertain despite years of experience with antiviral therapies, due to the dynamic immune status specific to CMV [1,3,33]. A recent worldwide survey on CMV prevention strategies in SOT found that universal prophylaxis was used in 90% of centers in D+/R− and in 50% in of R+ SOT with variable duration depending on the type of transplant, CMV serostatus, and induction immunosuppression [4^▪▪^].

Antiviral agents existing for prophylaxis are ganciclocir/valgancicovir and letermovir, recently approved for prevention of CMV in high-risk kidney SOT with very high safety profile [25^▪▪^]. However, late onset of CMV disease after discontinuation of prophylaxis requires new strategies to prevent this complication [34].

A recent randomized controlled trial concluded that among CMV D+/R− liver transplant recipients, the use of preemptive therapy, compared with antiviral prophylaxis, resulted in a lower incidence of CMV disease over 12 months in the preemptive group [35]. In a post hoc landmark analysis of long-term survival in this trial, long-term mortality was significantly lower in the preemptive therapy arm compared with the antiviral prophylaxis arm among 12-month survivors [36].

New stratification strategies through CMV-CMI, genetic polymorphisms and immune-monitoring may potentially improve tailored indication and duration of prophylaxis for CMV in SOT [9,10^▪▪^,^37^]. However, currently consensus guidelines do not provide clear recommendations related to timing of use and interpretations of these assays.

## TREATMENT OF REFRACTORY-RESISTANT CYTOMEGALOVIRUS INFECTION

The development of refractory and resistant CMV infection occurs relatively rarely but remains a major challenge and has been associated with increased morbidity and mortality in SOT [38]. CMV infection may fail to respond to commercially available antiviral therapies, with or without demonstrating genotypic mutations [39]. This lack of response has been termed “resistant/refractory CMV” and is a key focus of clinical trials of some investigational antiviral agents. Resistant CMV infection is defined as detection of a viral genetic mutation that decreases the susceptibility to one or more antivirals, whereas refractory CMV infection is characterized by persistent signs and symptoms of CMV disease and/or persistent CMV DNAemia that fails to improve, indicated by failure to attain a 1-log decline in viral load after 2 weeks of appropriately dosed antiviral therapy [39]. However, there is a significant gap between clinical practice and clinical trials definitions that does not allow to establish the true incidence of refractoriness to antivirals, with or without resistance in SOT population [40].

Most refractory CMV infections are due to resistant CMV with genotypic mutations that cause resistance to specific antiviral drugs, but other causes of refractory CMV include overimmunosuppression or inadequate drug dosing [2^▪▪^]. Definitive treatment of resistant CMV should be guided by the results of gene- resistance studies, including UL54, UL97, and UL56 mutations. Of interest that CMV genotypic resistance to antivirals has been independently associated with younger age, exposure to low levels of gancivlovir/valganciclovir, the recipients negative serostatus, and the occurrence of the infection on valganciclovir prophylaxis [38].

If drug resistance is suspected, alternative antiviral agents recommended by consensus guidelines are foscarnet, cidofovir and high dose ganciclovir (1). However, these therapies for refractory CMV infections in SOT are limited by toxicities. On the basis of its better efficacy and safety profile, oral maribavir is a preferred antiviral drug for treatment of selected patients with refractory CMV and those with genotypic resistance to ganciclovir, foscarnet, and cidofovir(2).

However, foscarnet still remains the preferred empiric therapy for refractory and resistant CMV disease affecting the central nervous system, including CMV encephalitis and retinitis, and for refractory CMV diseases with high viral loads, unless the virus is genotypically resistant to the drug [41]. These recommendations are based on knowledge of poor maribavir central nervous system penetration, limited available data on maribavir efficacy in refractory CMV diseases with high viral loads (only 6% of patients in SOLSTICE trial had high viral load), and on a significant concern for the risk of resistance development during and after treatment [31]. Due to foscarnet toxicity, transition to oral maribavir with a better safety profile is preferred, once the viral load has declined to low levels.

Adoptive immunotherapy, the transfer of CMV specific T-cells, offers a new approach in treatment of drug-resistant or refractory CMV infections, with early clinical trials and real life experience showing promising efficacy and safety [42].

## CONCLUSION

Despite advances in preventive strategies, CMV infection remains a significant challenge in SOT, being a driver of negative patient and allograft outcomes, especially in the setting of refractory or resistant CMV infections. CMV infection and disease management is improving with the accessibility of new diagnostic tests and with availability of new antiviral drugs. The optimal approach to the prevention and treatment of infection due to CMV remains uncertain, but cell-mediated immunity against CMV has the potential to improve tailored strategies. Letermovir may be as efficient as valganciclovir for preventing CMV disease with fewer myelotoxicity. Maribavir is now approved for treating refractory/resistant CMV infection. Further studies are still required to improve management of CMV in SOT.

## Acknowledgements

*None.*

### Financial support and sponsorship

*None.*

### Conflicts of interest

*P.A.G. has the following conflict of interest outside the submitted work: Consulting fees from Merck, Sharp & Dohme, Gilead Sciences, Takeda, Biotest, Allovir; member of speakers bureau for Merck, Sharp & Dohme, Gilead Sciences, Takeda.*

*M.P. reports receiving grants and personal fees from Pfizer, MSD, Menarini, Thermofisher and Dia Sorin outside the submitted work.*

## REFERENCES AND RECOMMENDED READING

Papers of particular interest, published within the annual period of review, have been highlighted as:

▪ of special interest
▪▪ of outstanding interest
